# Pulsed Field Ablation for Cardiac Arrhythmias: Industry Landscape and US Food and Drug Administration–approved Platforms (2025)

**DOI:** 10.19102/icrm.2026.17073

**Published:** 2026-07-15

**Authors:** Amar Ghaleb, Abimbola Kolawole, Usher Khan, Arfaat M. Khan

**Affiliations:** 1 Central Michigan University College of Medicine, Mount Pleasant, MI, USA; 2 Division of Cardiology, Henry Ford Health, Detroit, MI, USA

**Keywords:** Atrial fibrillation, cardiac electrophysiology, catheter ablation, FDA-approved ablation systems, hybrid RF–PFA

## Abstract

Pulsed field ablation (PFA) has rapidly advanced from an experimental modality to a front-line therapy for catheter ablation of atrial fibrillation (AF). Unlike thermal approaches such as radiofrequency (RF) or cryoablation, PFA relies on irreversible electroporation, producing rapid and selective myocardial lesions with minimal risk of collateral injury. This review examines the contemporary landscape of PFA technology, with emphasis on recent United States Food and Drug Administration (FDA) approvals that have expanded PFA from single-energy, single-shot systems toward focal and hybrid ablation strategies. Particular focus is placed on FDA-approved platforms, including PulseSelect™ (Medtronic, Minneapolis, MN, USA), FARAPULSE™ (Boston Scientific, Marlborough, MA, USA), and VARIPULSE™ (Biosense Webster, Diamond Bar, CA, USA), as well as next-generation systems such as the Affera Sphere-9™ dual-energy RF–PFA platform (Medtronic) and the Volt™ focal PFA system (Abbott, Chicago, IL, USA). Differences in catheter design, energy delivery, workflow, mapping integration, regulatory labeling, and clinical outcomes are reviewed. By contextualizing these platforms within evolving ablation strategies, this review highlights a second evolutionary phase of PFA focused on procedural flexibility, lesion durability, and integration with established electrophysiology (EP) workflows, with implications for future AF management and EP lab practice.

## Introduction

Atrial fibrillation (AF) is the most common sustained arrhythmia and a major driver of cardiovascular morbidity and health care services use. Catheter ablation traditionally employs thermal energy—radiofrequency (RF) ablation through resistive and conductive heating and cryoablation through freezing and ice crystal formation. These methods carry risks of collateral damage, including atrioesophageal fistula, pulmonary vein stenosis, and phrenic nerve palsy. Pulsed field ablation (PFA) differs fundamentally. It delivers ultra-short, high-voltage electrical pulses that induce irreversible electroporation, selectively disrupting cardiomyocyte membranes while sparing surrounding tissues such as the esophagus, phrenic nerves, and pulmonary veins.^1^ This nonthermal and tissue-selective mechanism enables faster lesion creation and greater safety and has fueled rapid adoption following multiple United States (US) Food and Drug Administration (FDA) approvals between 2023 and 2025.

## Methods

This review surveyed peer-reviewed publications, including clinical trials, registries, and review articles, as well as publicly available regulatory documents such as FDA premarket approvals, summaries of safety and effectiveness data, and manufacturer technical documentation. Literature was identified through searches of PubMed and major cardiovascular journals, and regulatory information was obtained directly from the FDA database. Sources published through February 9, 2026, were included.

Focus was placed on device architecture, energy delivery, procedural workflow, mapping integration, clinical safety and efficacy outcomes, and on-label regulatory indications, with labeling and performance claims interpreted in the context of available clinical and regulatory evidence. This article is a review of published literature and does not involve new human subjects research; therefore, ethical approval and informed consent were not required.

## Findings

### Industry landscape (2025)

At the time of writing, three dedicated single-energy PFA platforms are FDA-approved in the US: PulseSelect™ (Medtronic, Minneapolis, MN, USA), FARAPULSE™ (Boston Scientific, Marlborough, MA, USA), and VARIPULSE™ (Biosense Webster, Diamond Bar, CA, USA).^2–4^ In addition, two next-generation systems received FDA approval between 2024 and 2025 that materially alter the competitive and technological landscape: the Affera Sphere-9™ dual-energy (RF + PFA) system (Medtronic) and the Volt™ PFA platform (Abbott, Chicago, IL, USA).

Sphere-9™ represents the first FDA-approved catheter capable of seamlessly delivering both RF and pulsed field energy within a single focal lattice-tip design, enabling tailored lesion creation for pulmonary vein isolation (PVI), linear lesions, and non-pulmonary vein targets. Meanwhile, Volt™, approved in 2025, expands the US market to a fourth manufacturer and emphasizes focal, contact force-guided PFA lesion delivery with conventional workflow familiarity.

These approvals mark a transition from single-shot PFA systems toward convergent and hybrid ablation strategies, with important implications for durability, operator learning curves, and the future role of thermal energy.^5–9^

Growth in PFA trials and approvals since 2019 is shown in **Figure 1**.

**Figure 1:**
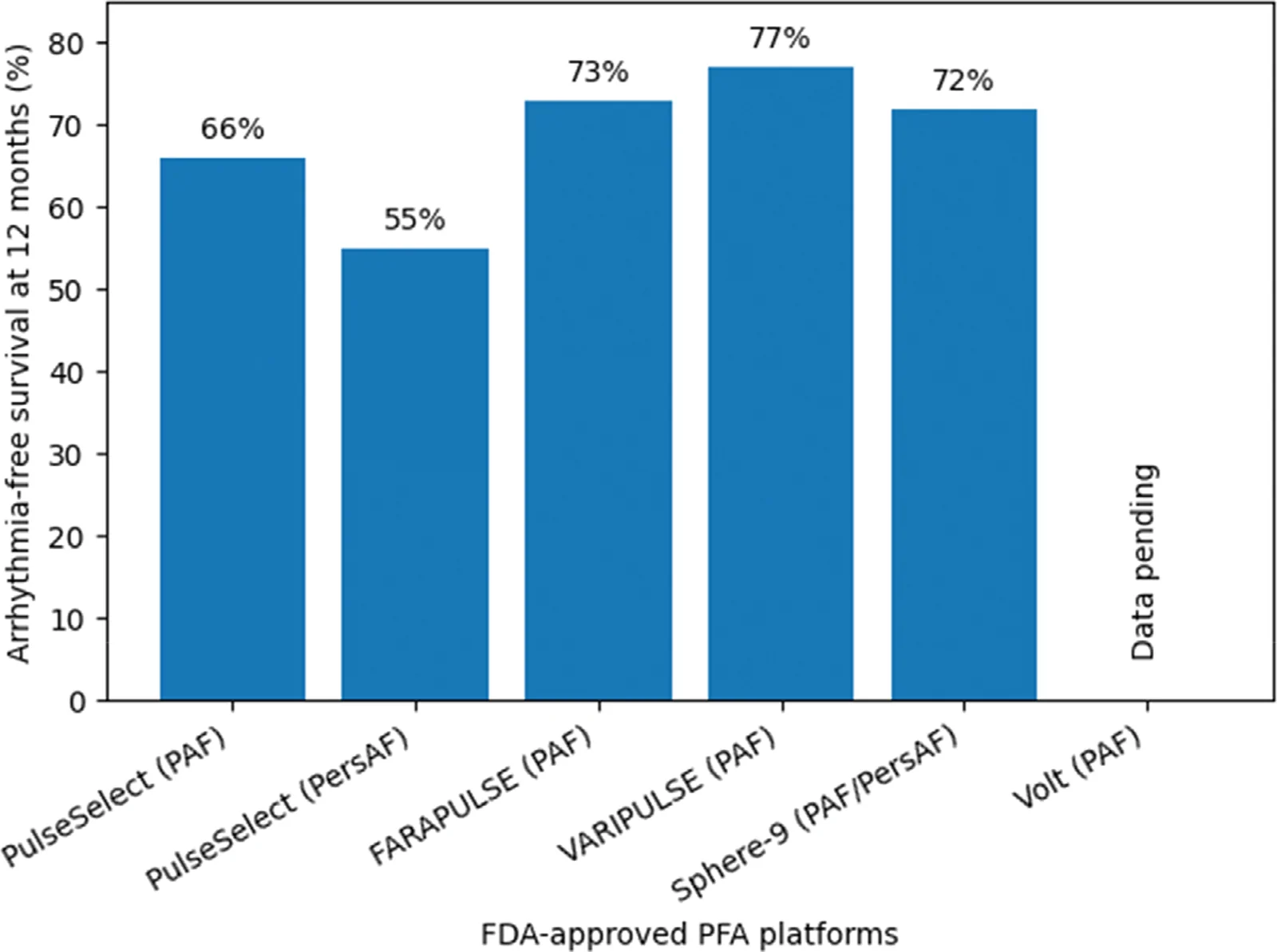
Temporal growth in pulsed field ablation (PFA) clinical investigation and regulatory adoption between 2019 and 2025. The figure illustrates the increasing number of prospective clinical trials, pivotal studies, and major regulatory approvals for PFA systems over time, reflecting the rapid transition of electroporation-based ablation from early feasibility studies to widespread clinical implementation. Key inflection points correspond to first-in-human studies, pivotal trials supporting US Food and Drug Administration approvals, and the subsequent expansion to hybrid and focal PFA platforms.

### FDA-approved platforms: technology, technique, and methods

PulseSelect™ employs a 25-mm loop catheter with nine electrodes delivering biphasic, bipolar energy to minimize skeletal muscle capture. The workflow centers on single-shot PVI with catheter rotation. The PULSED-AF IDE (“Pulsed Field Ablation for Atrial Fibrillation Investigational Device Exemption”) trial reported approximately 66% 1-year arrhythmia-free survival for paroxysmal AF and 55% for persistent AF.^10^ The US label includes both paroxysmal and persistent AF (<1 year).^11^

FARAPULSE™ consists of the FARAWAVE pentaspline catheter and FARASTAR generator, integrated with FARAVIEW on OPAL HDx for map-and-ablate workflows.^12^ The ADVENT (“The FARAPULSE ADVENT PIVOTAL Trial PFA System vs. SOC Ablation for Paroxysmal Atrial Fibrillation”) trial demonstrated the noninferiority of PFA to thermal ablation for paroxysmal AF with fewer esophageal and pulmonary vein complications.^13^ In 2025, the FDA expanded labeling to include posterior wall ablation for persistent AF.^3^

VARIPULSE™ uses a variable-loop circular catheter with 10 irrigated electrodes and integrates with CARTO™ 3 via the TRUPULSE generator. Studies (admIRE [“Assessment of Safety and Effectiveness in Treatment Management of Atrial Fibrillation with the Biosense-Webster Irreversible Electroporation Ablation System”], inspIRE [“Study for Treatment of Paroxysmal Atrial Fibrillation by Pulsed Field Ablation System with Irreversible Electroporation”]) reported 74%–80% arrhythmia-free survival at 12 months.^14^ The US label currently includes PVI for paroxysmal AF.^4^

Sphere-9™ employs a focal lattice-tip catheter capable of delivering both irrigated RF energy and PFA via a single platform integrated with the EnSite X™ mapping system. FDA approval in 2024 followed the SPHERE Per-AF (“Safety and Performance Assessment of the Sphere-9 Catheter and the Affera Mapping and RF/PF Ablation System to Treat Atrial Fibrillation”) and RADIANCE (“A Study of the Recor Medical Paradise System in Clinical Hypertension”) trials, which demonstrated high acute PVI durability and favorable safety across paroxysmal and persistent AF.

The hybrid capability differentiates Sphere-9™ from single-energy PFA systems by allowing operators to apply RF for areas requiring deeper or more contiguous lesions (eg, mitral isthmus, cavotricuspid isthmus) while leveraging PFA’s tissue selectivity near the esophagus or phrenic nerve. Early post-approval registries suggest procedural flexibility and reduced reliance on adjunctive catheters.^8,9^

Finally, the Abbott Volt™ system received FDA approval in 2025, transitioning from earlier CE-mark experience to US clinical adoption. Volt™ uses a focal PFA catheter designed for point-by-point lesion deployment with contact force sensing and integration into Abbott’s EnSite™ mapping ecosystem.

Unlike basket-based single-shot platforms, Volt™ emphasizes workflow continuity for operators accustomed to focal RF ablation, potentially lowering the barrier to adoption while preserving the myocardial selectivity of irreversible electroporation. Early clinical studies report high acute PVI success and low complication rates, though long-term durability data remain forthcoming.^6,15^

A brief comparative analysis of these technologies is presented in **Table 1**. **Table 2** provides a more detailed comparison of catheter form factors and mapping integration among the different available platforms.

**Table 1:** Comparative Analysis of Available FDA-approved Pulsed Field Ablation Platforms

Attribute	PulseSelect™	FARAPULSE™	VARIPULSE™	Sphere-9™	Volt™
Energy	Biphasic bipolar	Multipulse bipolar	Biphasic	RF + PFA (dual)	PFA (focal)
Catheter	25-mm loop	Pentaspline	Variable loop	Lattice-tip focal	Focal CF enabled
Mapping	Standalone	OPAL HDx	CARTO 3	EnSite X™	EnSite™
FDA label	PAF + PersAF	PAF + PWA	PAF	PAF + PersAF	PAF
Strategic role	Single shot	Single shot	Mapping centric	Hybrid durability	RF-like workflow

*Abbreviations:* CF, contact force; PAF, paroxysmal atrial fibrillation; PersAF, persistent atrial fibrillation; PWA, posterior wall ablation.

**Table 2:** Comparison of Catheter Form Factors and Mapping Integration Among FDA-approved Pulsed Field Ablation Platforms (United States, 2025)

Platform	Catheter Geometry/Form Factor	Mapping Integration	Labeling (United States, 2025)
PulseSelect™	25-mm circular loop, 9 electrodes	Standalone/Affera	Paroxysmal and persistent AF (<1 year)
FARAPULSE™	Pentaspline (basket/flower) configuration	FARAVIEW + OPAL HDx	Paroxysmal AF + posterior wall (PersAF)
VARIPULSE™	Variable-diameter circular loop, 10 electrodes	CARTO 3	Paroxysmal AF
Sphere-9™	Focal lattice-tip catheter (dual-energy RF + PFA)	EnSite X™	Paroxysmal and persistent AF
Volt™	Focal contact force–enabled PFA catheter	EnSite™	Paroxysmal AF

*Abbreviations:* AF, atrial fibrillation; FDA, US Food and Drug Administration; PersAF, persistent atrial fibrillation; PFA, pulsed field ablation; RF, radiofrequency. Note that FDA-approved PFA platforms demonstrate substantial heterogeneity in catheter geometry, mapping integration, and regulatory labeling. Early single-shot platforms emphasize circular or multielectrode pulmonary vein isolation strategies, whereas newer focal and hybrid systems enable point-by-point lesion delivery and dual-energy ablation. These differences have important implications for procedural workflow, lesion durability, and applicability to persistent AF substrates.

### Clinical outcomes and safety

Across randomized trials, post-approval registries, and real-world observational data, PFA demonstrates efficacy comparable to, and in some studies numerically favorable to, thermal ablation, with approximately 70%–80% arrhythmia-free survival at 12 months following PVI for paroxysmal AF. Reported 12-month arrhythmia-free survival rates across platforms is shown in **Figure 2**. The durability of PVI has been consistently high, with acute isolation rates exceeding 95% across FDA-approved platforms, reflecting the rapid and homogeneous lesion formation achievable with irreversible electroporation.^16^

**Figure 2:**
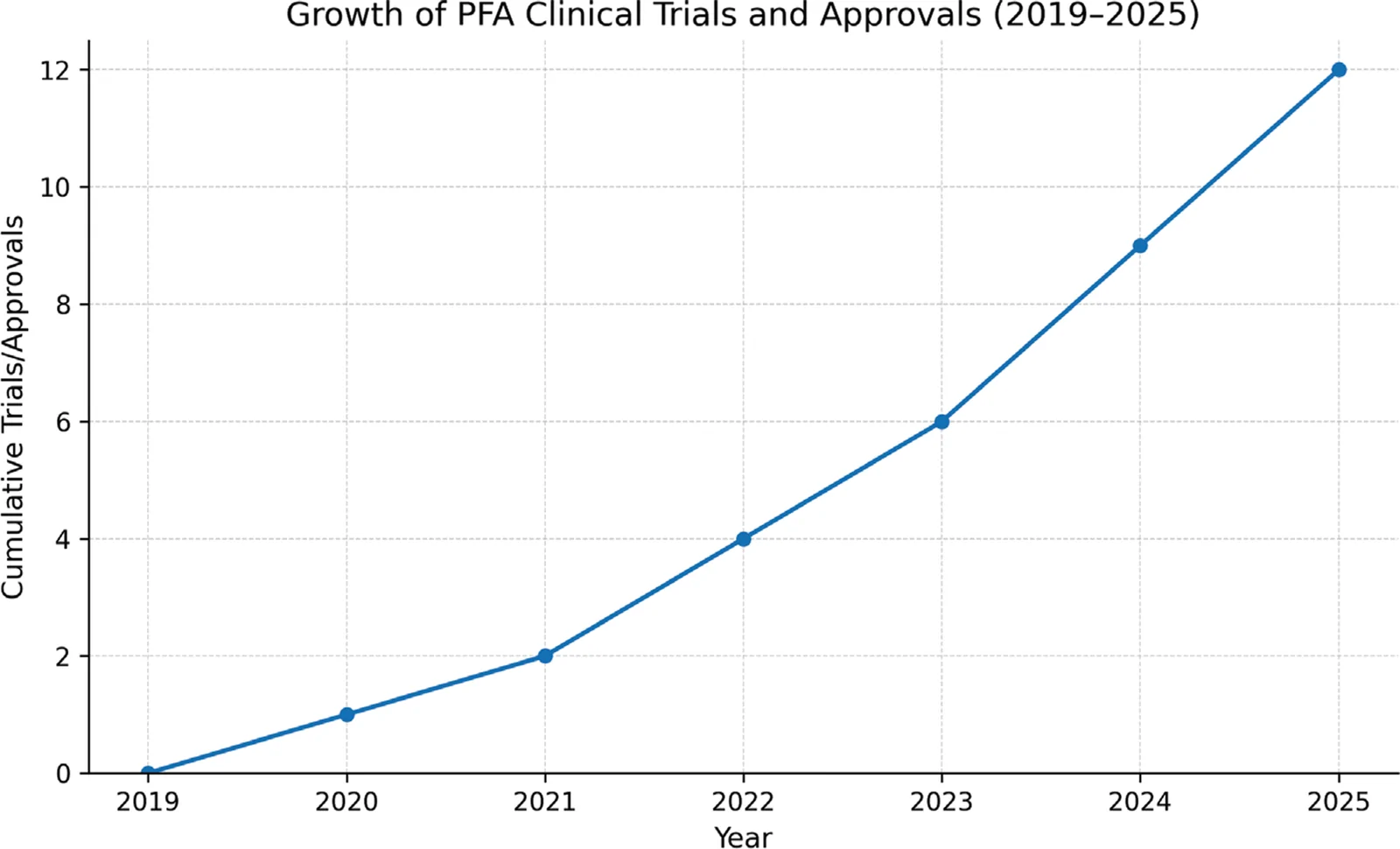
Reported 12-month arrhythmia-free survival following pulmonary vein isolation using US Food and Drug Administration–approved pulsed field ablation platforms. Data are derived from pivotal trials, prospective registries, or early post-approval studies and are shown for descriptive comparison purposes rather than direct head-to-head evaluation. Differences in patient populations, study design, and follow-up methodology limit cross-platform comparability. Hybrid and focal systems approved more recently, including Sphere-9™ and Volt™, have less mature long-term outcome data. *Abbreviations:* PAF, paroxysmal atrial fibrillation; PersAF, persistent atrial fibrillation.

A defining advantage of PFA is its favorable safety profile resulting from myocardial tissue selectivity. Unlike RF and cryoablation, PFA has demonstrated markedly reduced rates of esophageal injury, pulmonary vein stenosis, and phrenic nerve palsy, complications that historically limited thermal ablation strategies.^13^ Endoscopic studies following PFA have shown minimal to no esophageal thermal injury, and large registries report an absence of atrioesophageal fistula, one of the most feared complications of AF ablation.

The safety of PFA at scale was further confirmed in the MANIFEST-17K (“Multinational Assessment of Novel Pulsed Field Ablation Studies”) registry, which evaluated outcomes in >17,000 patients and demonstrated exceptionally low rates of major adverse events, including tamponade, stroke, and mortality.^13^ Phrenic nerve injury was rare and typically transient, and no clinically significant pulmonary vein stenosis was observed, reinforcing the anatomic selectivity of electroporation-based lesions.

Despite these advantages, cerebrovascular events remain an area of ongoing scrutiny. Microembolic signals have been detected during PFA delivery, and periprocedural stroke and transient ischemic attack, although uncommon, underscore the importance of meticulous anticoagulation management, optimized catheter manipulation, and continued post-market surveillance.^13^ Additionally, muscle capture and transient diaphragmatic stimulation may occur, though advances in biphasic and bipolar waveform design have substantially mitigated these effects.

Overall, the accumulating body of evidence supports PFA as a highly effective and safer alternative to thermal ablation, particularly for PVI.^17^ As indications expand to include posterior wall ablation and persistent AF, long-term comparative data will be critical to define durability, stroke risk, and outcomes beyond 1 year; however, current data strongly favor PFA as a transformative advancement in catheter-based arrhythmia therapy.^7^ Importantly, hybrid RF–PFA systems such as Sphere-9™ may mitigate current limitations of single-energy PFA in achieving durable linear lesions and non-pulmonary vein targets, potentially redefining ablation strategies for persistent AF and complex substrates.^8,9^

### Future directions

Future applications of PFA include expanded strategies for persistent AF, such as posterior wall ablation, as well as investigation into ventricular arrhythmias and the continued development of dual-energy (RF–PFA) systems. Emerging technologies, including nanosecond PFA, offer the potential for increasingly precise lesion creation, though clinical validation remains ongoing.

From a health systems perspective, PFA may reduce total health care services use by shortening procedural times, lowering complication rates, and decreasing the need for repeat interventions. While per-device costs are currently higher than those of conventional thermal technologies, procedural efficiency and improved safety profiles could favorably influence electrophysiology laboratory economics over time.

As clinical experience and indications expand, RF and cryoablation are likely to remain integral components of arrhythmia management, with hybrid and convergent platforms increasingly enabling tailored energy selection based on anatomic and substrate-specific considerations. Collectively, these trends suggest that PFA represents a durable advancement in catheter ablation technology with the potential to reshape electrophysiology practice, pending confirmation from long-term comparative and outcomes-based studies.

## Conclusion

PFA has moved from concept to clinical reality in <5 years. PulseSelect™, FARAPULSE™, and VARIPULSE™ each offer distinct advantages: broad labeling, robust randomized evidence, and deep mapping integration, respectively. The nonthermal, selective mechanism of PFA provides a compelling safety and efficiency profile, positioning it to redefine catheter ablation and reshape the future of electrophysiology. The recent FDA approvals of hybrid and focal PFA platforms (Sphere-9™ and Volt™) mark a second evolutionary phase of PFA, shifting the field from safety validation toward optimization of lesion durability, procedural flexibility, and workflow convergence. By contextualizing these approvals within evolving ablation strategies, this review highlights how PFA is transitioning from a disruptive technology to an adaptable therapeutic framework for increasingly complex AF substrates.
